# Supplementary material for: Spatial Transcriptomic Analysis of Surgical Resection Specimens of Primary Head and Neck Squamous Cell Carcinoma Treated with Afatinib in a Window-of-Opportunity Study (EORTC90111-24111)
Source: Int J Mol Sci. 2025 Feb 20;26(5):1830. doi: 10.3390/ijms26051830 (PMC11898532; doi:10.3390/ijms26051830)
Supplement: Supplementary file 1 [file ijms-26-01830-s001.zip › Supplementary figures.docx]

**Supplementary figures**

**
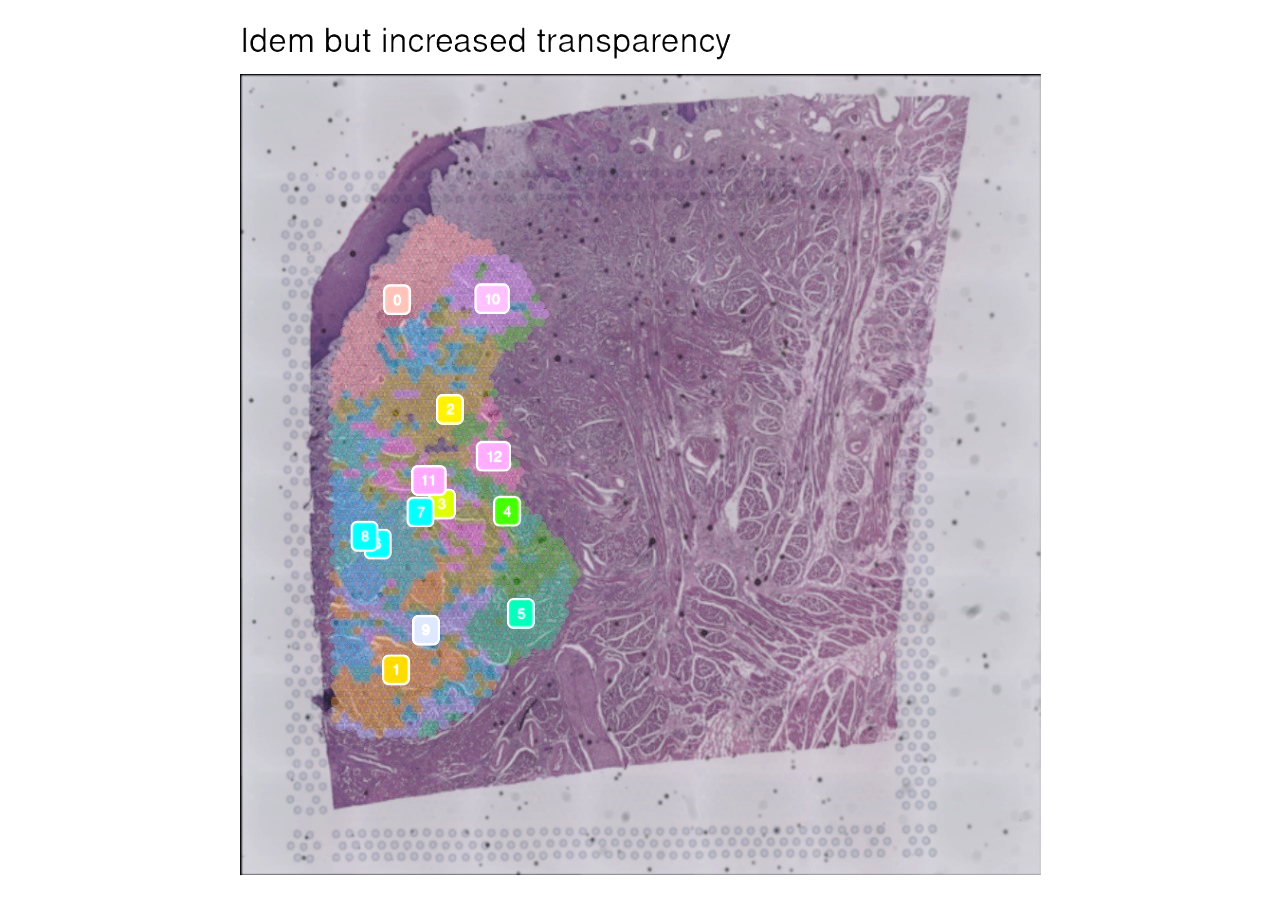

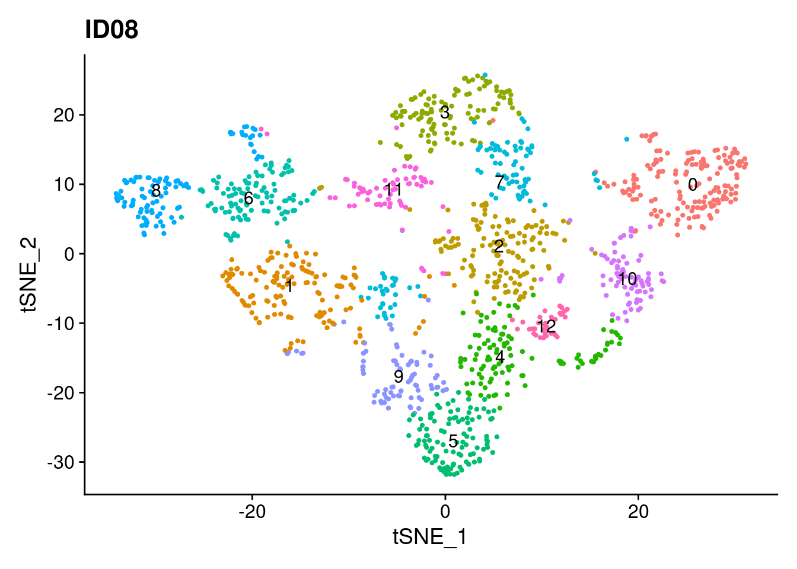
Figure S1.** t-SNE (t-distributed stochastic neighbor embedding) dimension reduction of spots included in the region of interest of sample ID08.

**Figure S2.** Clusters spatially represented on the H&E slide of patient ID08.

H&E: hematoxylin and eosin

**
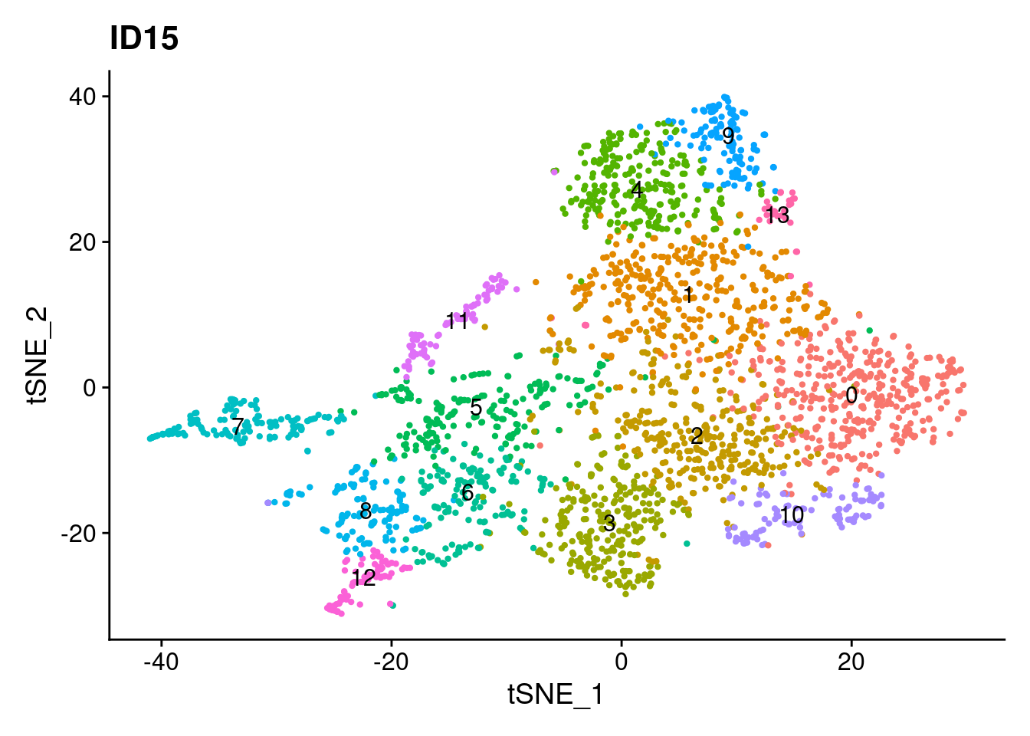
Figure S3.** t-SNE (t-distributed stochastic neighbor embedding) dimension reduction of spots included in the region of interest of sample ID15.

**
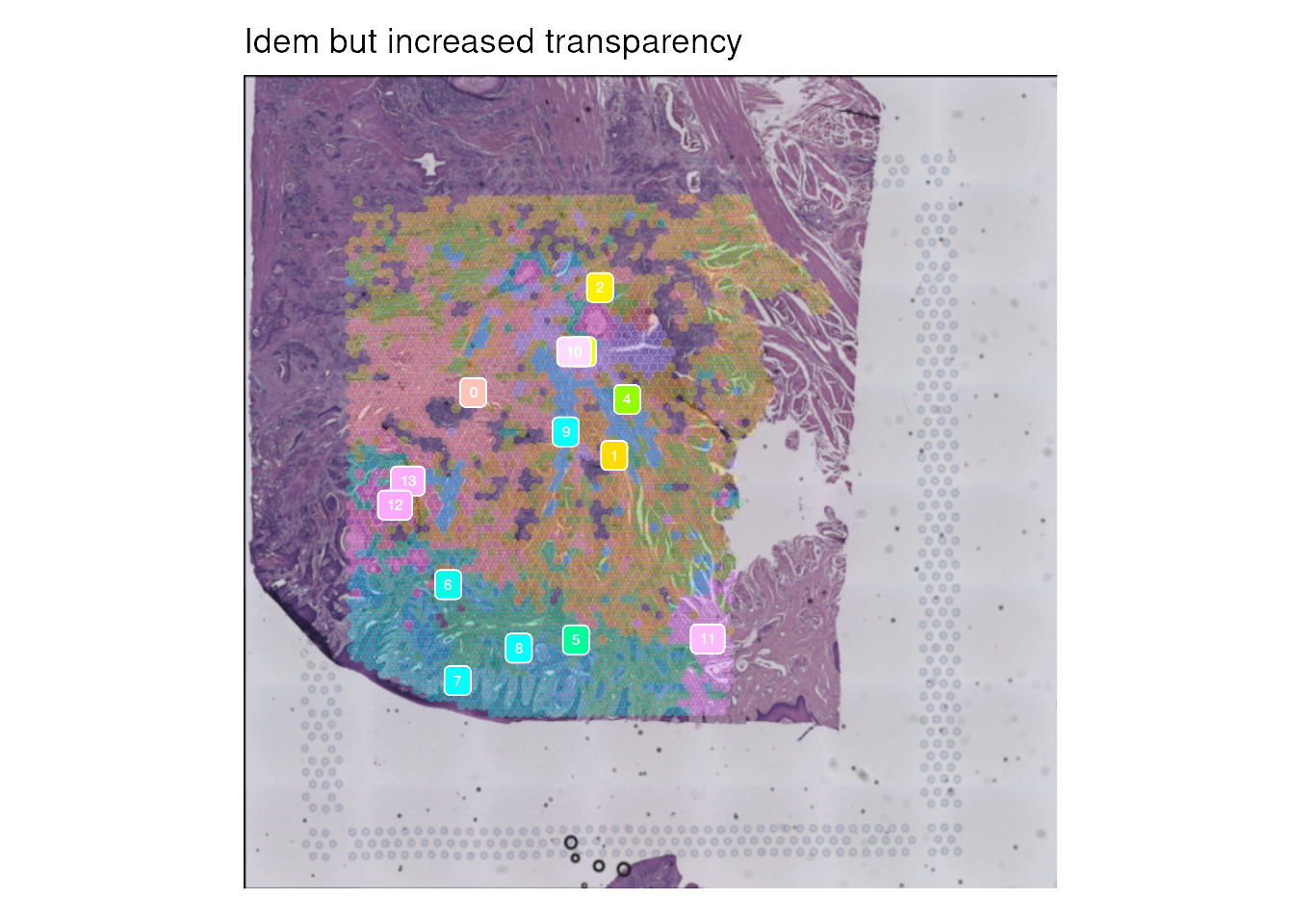
Figure S4.** Clusters spatially represented on the HE slide of patient ID15.

H&E: hematoxylin and eosin

**
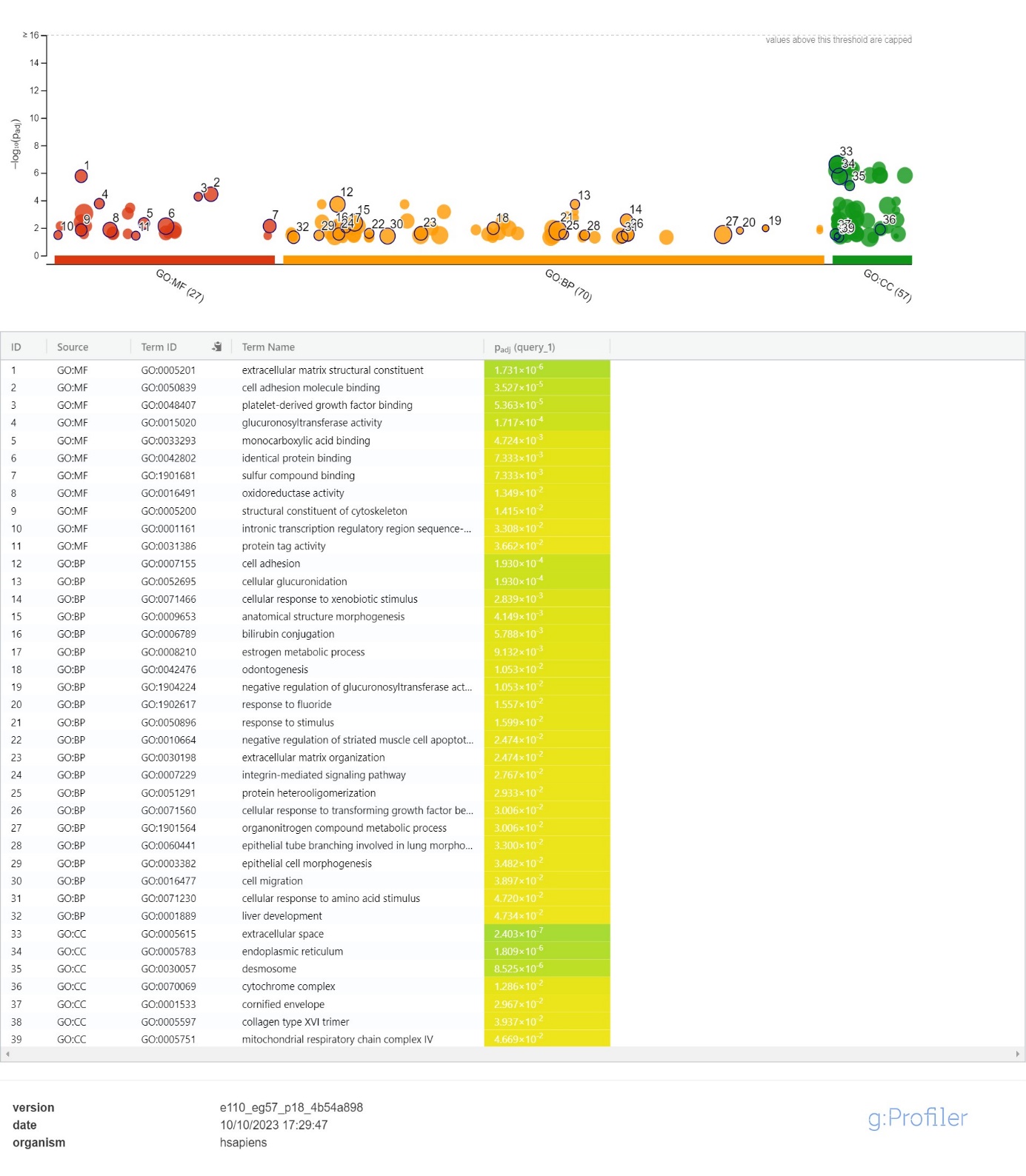
Figure S5.** Enrichment analysis of 123 genes upegulated in common between differential exrepssion analyses of tumor nodules in patient ID30.

**
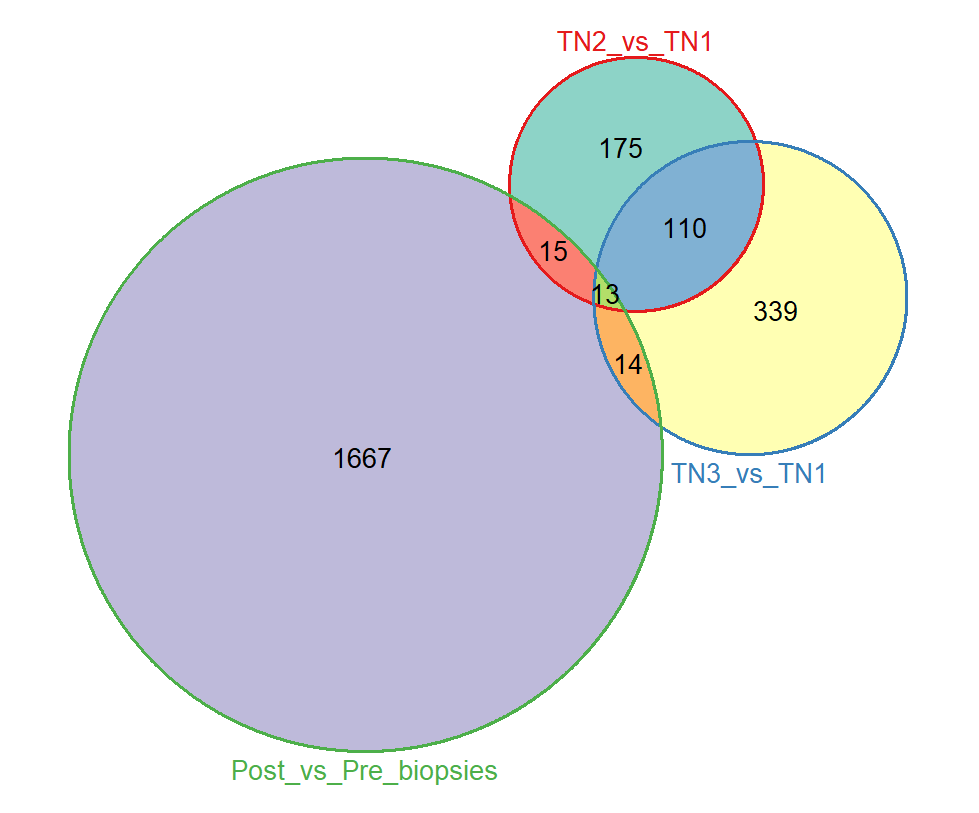
Figure S6.** Venn plot showing upregulated genes in common between the differential analyses in tumor nodules and in post vs pre-afatinib tumor biopsies.

TN: tumor nodule

**
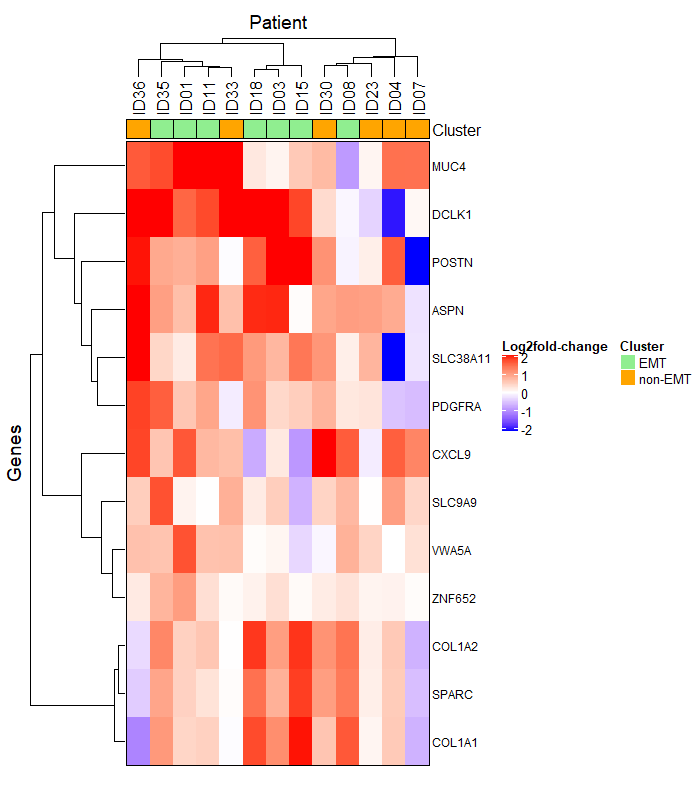
Figure S7.** Heatmap highlighting the log2-fold-change expression of the 13 upregulated genes in common between the differential analyses in tumor nodules in patient ID30 and in post vs pre-afatinib tumor biopsies in patients from the EORTC90111 study^11^. The Log2fold-change represents the log2 of the normalized gene expression in tumor biopsies after afatinib minus the log2 of the normalized expression of these same genes before afatinib in tumor biopsies. The clusters already described in Beyaert et al.^11^ are highlighted in light green (EMT cluster) and orange (non-EMT cluster).
